# Lifting without Seeing: The Role of Vision in Perceiving and Acting upon the Size Weight Illusion

**DOI:** 10.1371/journal.pone.0009709

**Published:** 2010-03-15

**Authors:** Gavin Buckingham, Melvyn A. Goodale

**Affiliations:** Centre for Brain and Mind, University of Western Ontario, London, Ontario, Canada; Kyushu University, Japan

## Abstract

**Background:**

Our expectations of an object's heaviness not only drive our fingertip forces, but also our perception of heaviness. This effect is highlighted by the classic size-weight illusion (SWI), where different-sized objects of identical mass feel different weights. Here, we examined whether these expectations are sufficient to induce the SWI in a single wooden cube when lifted without visual feedback, by varying the size of the object seen prior to the lift.

**Methodology/Principal Findings:**

Participants, who believed that they were lifting the same object that they had just seen, reported that the weight of the single, standard-sized cube that they lifted on every trial varied as a function of the size of object they had just seen. Seeing the small object before the lift made the cube feel heavier than it did after seeing the large object. These expectations also affected the fingertip forces that were used to lift the object when vision was not permitted. The expectation-driven errors made in early trials were not corrected with repeated lifting, and participants failed to adapt their grip and load forces from the expected weight to the object's actual mass in the same way that they could when lifting with vision.

**Conclusions/Significance:**

Vision appears to be crucial for the detection, and subsequent correction, of the ostensibly non-visual grip and load force errors that are a common feature of this type of object interaction. Expectations of heaviness are not only powerful enough to alter the perception of a single object's weight, but also continually drive the forces we use to lift the object when vision is unavailable.

## Introduction

In the size-weight illusion (SWI), smaller objects feel heavier than larger objects with the same weight and surface properties [1]. Despite over 100 years of research, the underlying cause of this striking illusion is still unknown. A compelling suggestion is that participants lifting SWI stimuli erroneously predict the large block will weigh more than the small block, and, as a consequence, grip and lift it with more force than they use on the small block. Individuals lifting SWI stimuli would experience sensory feedback that differs from what they expected, and this mismatch between expectation and perception may cause the illusory differences in weight between the various identically-weighted objects [2]. Recently, however, researchers have undermined this sensorimotor version of the ‘expectation theory’ of the SWI by showing rapid adaptation of fingertip forces to each object's mass, independent of heaviness judgments [3], [4]. Participants continue to report the smaller block as feeling heavier than the larger block, long after their initial lifting errors (and sensorimotor mismatch) have been implicitly corrected.

The rejection of the sensorimotor hypothesis has led to an increased interest in the role that individuals' cognitive expectations of heaviness play in the SWI. Individuals expect larger items to weigh more than smaller items, reflecting the statistics of the natural world [5], and the illusion stems from the failure to meet this expectation in the stimulus set (where all the objects have the same mass). Unfortunately for proponents of this theory, the illusion itself is not cognitively penetrable – it has long be known that the illusion persists even well after subjects have been told the stimuli all have the same mass [6]. Furthermore, no researchers have been able to induce a comparable weight illusion with expectations alone. While it has been demonstrated that the availability of vision during lifts of SWI stimuli contributes (independently from haptics) to experiencing the ‘full-strength’ illusion [7], it is far from clear whether or not expectations add to, or even cause, the illusory experience. The few studies that have investigated weight illusions in a more cognitive context than the SWI [8], [9] have all confounded expectations of heaviness with continuous visual input during the task. This is problematic for determining the ‘pure’ role of cognitive expectations in weight illusions, as the integration of visual and proprioceptive information has been proposed as a causal factor in the SWI [10]. In fact, Masin and Crestoni [11] demonstrated that continuous feedback of the lifted object is necessary to experience the SWI. Participants in their experiment lifted bottles by pulling down on strings, rigged with a pulley system. When these ‘lifts’ were performed without vision of the bottles, participants did not experience a size-weight illusion (which of course they did experience when they could see the bottles rise into the air). Their experiment has remained the most compelling critique of the cognitive expectation theory of the SWI, since common sense informs us that relevant expectations of how heavy an object will be should persist after even the briefest cue to size.

In the current study, we aimed to investigate if Masin and Crestoni's [11] refutation of the cognitive expectation theory is applicable to a more natural lifting scenario, where objects are lifted with a precision grip. To this end, we compared individuals' perception of SWI stimuli in a classic version of the task (where participants lift small, medium, and large objects of identical mass, with full vision of the task) with an ‘expectation-only’ variant. In this alternate SWI task, participants lifted the same medium-sized cube *without vision*, which was secretly placed in front of them following a brief glimpse of a different-sized cube. We predicted that seeing a small cube would make them expect an easy lift, while seeing a large cube would prime them for a more difficult lift, and these expectations would be powerful enough not only to make them lift the medium block with respectively lesser or greater force than necessary, but also to alter the perception of the lifted object's weight. By measuring the fingertip forces used to lift the object in this expectation-only variant of the classic SWI task, we also aimed to examine how vision contributes to the fast and accurate scaling of grip and load force to real, rather than perceived, object mass.

## Results and Discussion

**Figure 1:**
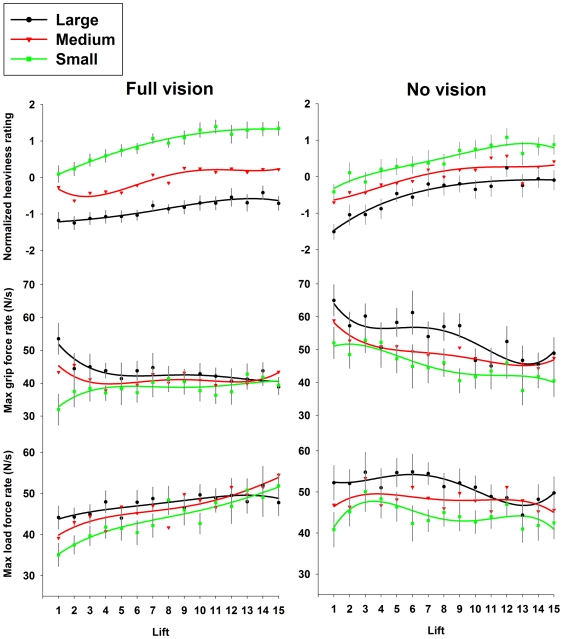
Fingertip forces and perceptual ratings. The perceptual and kinetic measures for the full-vision, classic SWI task (left side) where participants lifted 3 blocks with different sizes, but identical masses, and the no-vision task (right side), where participants lifted the same block throughout the experiment without vision, varying only the size of the block that was seen before the lift. Error bars represent standard error of the mean of the difference between the small and large blocks.

In the full-vision experiment, participants reported that the identically-weighted cubes had different weights – the SWI (main effect of size; F_(2,38)_ = 186.50,p<.001; Figure 1a [top]). While participants reported that all the cubes tended to feel heavier the course of the experiment, presumably due to participants' fatigue with repeated lifts [3], the strength of the illusion did not decrease. Consistent with previous reports [2], [3], participants were able to quickly adjust their grip and load force rates from the *expected* weight of each object to the *actual* mass of each object (size by trial interaction; F_(28,532)_ = 1.71,p<.05 and F_(28,532)_ = 1.66,p<.05 respectively; Figure 1a [middle and lower]). The errors made during the first few lifts of each cube (the application of a higher rate of force to the large block than the small block) were quickly corrected.

When participants lifted the single (medium-sized) cube in the no-vision condition, their estimates of its weight varied as function of what they saw before the lift (main effect of size; F_(2,36)_ = 25.34,p<.001 – Figure 1b [top]). In other words, if they had seen the large cube prior to the lift, the medium-sized cube that they eventually lifted felt lighter than it did when they had seen the small cube. The magnitude of this novel variant of the SWI did not diminish over time. To our knowledge, this is a unique demonstration of a weight illusion without continuous visual or haptic experience of an object's size or material during a lift. In contrast to the few studies which have made the same object feel different weights while presenting altered visual feedback of the relevant movement [12], [13], our illusion is induced by expectations alone. It should be noted that the magnitude of this expectation-only illusion appears to be somewhat smaller than the classic SWI observed in the full-vision condition. This discrepancy does not undermine our conjecture that expectations are sufficient to induce the SWI, but instead points to additive effects that visual feedback [7], [10] and the haptic sensation of the rotational inertia during a lift [14], [15] can have on the illusory experience.

Interestingly, the rates of forces that participants applied to the cube in the no-vision task over repeated lifts were substantially different to the forces applied in the normal, full-vision task. The grip force rates for all objects tended to be larger than when participants had vision – presumably a strategic response to increase the safety margin (the ratio of grip force to load force) due to uncertainty about the quality of contact with the grasp handle. Interestingly, rather than adapting their fingertip forces to the actual mass of each object, participants persisted in applying forces in line with their expectations of heaviness - no size by trial interaction was observed with grip force rate or load force rate (p = .62 and p = .84 respectively; see Figure 1b [middle and lower]). Thus, even though the same block was lifted across all trials, seeing the largest object before the lift always made participants grip and lift with a higher rate of force than when they saw the smaller block. It would appear that not viewing the lift itself interferes with the motor system's ability to either detect its initial error, or learn more appropriate grip and load forces for an object of a particular mass. We find this result particularly surprising, given that the underestimation and overestimation errors associated with object lifting are thought to be detected through somatosensation alone [16]. Of course, had the experiment progressed over enough trials, some scaling of the fingertip forces may have eventually taken place. However, it is clear that the rate of this scaling is dramatically affected by visual feedback.

It would seem that the role of vision in detecting and correcting errors in predictive fingertip forces has been widely underestimated. It is not, however, immediately obvious at what stage during the lift that vision is particularly crucial for the detection of these errors. Perhaps the comparison between expected lift off time and actual lift off time is monitored primarily by vision, rather than touch. Alternatively, visual feedback could be crucial for detecting that a lift that was too fast or slow (i.e., an unsafe/inefficient rate of change of load force), which may be synergistically yoked with the non-visual grip forces [17]. Another possibility, altogether unrelated to the detection of error, is that vision may be a base requirement for ‘normal’ skilled behavior of this type, and the systems controlling grip and load forces switch to a different, expectation-guided mode when multimodal stimulus combinations are unavailable. It is even possible that the five-second delay between seeing and lifting the object, rather than continuous visual feedback, is the crucial factor determining the participants' inability to scale their fingertip forces, analogous to the distinction between the proposed roles of the dorsal and ventral visual streams in delayed size-contrast illusion grasping tasks [18].

The current study provides a unique demonstration of the influence that cognitive expectations and vision of our actions have on our perception of weight and scaling of our fingertip forces. Expectations of heaviness are sufficient to induce the SWI, and are even powerful enough to alter the apparent weight of a single object. But, without the opportunity of witnessing our lifts, these expectations persist in driving fingertip forces over repeated encounters with an object.

## Materials and Methods

### Sample

Thirty-nine right-handed university undergraduates lifted either different SWI-inducing objects with full-vision (n = 19; 5 male, 14 female; mean age = 27.8 years, SD = 10.0), or the same object without vision (n = 20; 3 male, 17 female; mean age = 23.2 years, SD = 2.8). Participants gave written informed consent prior to testing and all procedures were approved by the University of Western Ontario Research Ethics Board and are also in accordance to the standards outlined in the 1964 Declaration of Helsinki.

### Full-vision task

**Figure 2:**
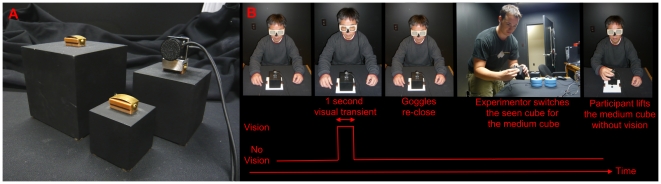
Stimuli and task. **A**: The stimuli used to elicit the SWI and **B**: the task in the no-vision (expectation only) condition.

Participants sat in front of a table wearing closed PLATO shutter goggles. The experimenter attached a plastic handle containing a pair of force transducers to one of the SWI stimuli (700g black hollow wooden cubes, weighted with varying amounts of lead: small [5 cm^3^], medium [7.5 cm^3^], or large [10 cm^3^] – see Figure 2a), and placed it on the table. The goggles opened at the same time as an auditory go cue, at which point participants reached out and lifted the object with their dominant hand's thumb and index finger on the force transducer handle, held it aloft for 3 seconds, and returned it to the starting position.

### No-vision task

In the no-vision task, the same stimuli as in the full-vision task were seen by the participants. However, instead of letting the participants lift these cubes with full vision, the shutter goggles opened for only one second, giving them a brief view of the (small, medium, or large) cube on the table. The goggles then closed, at which point the experimenter replaced the ‘seen object’ with the medium-sized cube - the only object lifted by participants in this condition. Participants were unaware of this change. After five seconds had elapsed (the time needed for the experimenter to discreetly replace the ‘seen block’ with the medium-sized ‘lifted block’), an auditory cue signaled to participants that they should lift the object as described above, while the goggles remained closed (see Figure 2b). It is important to emphasize that participants lifted only the same medium-sized cube in the no-vision task – each lift had identical torques, was started from identical heights, and required identical absolute levels of force to overcome the effects of gravity. All that changed across lifts was what the participant *saw before they lifted*, meaning that any changes in perceived weight and/or fingertip forces from one lift to the next must have been due to participants' expectations.

### Analysis

After every lift, when the object was returned to the tabletop, participants gave an unconstrained number representing how heavy the cube that they had just lifted felt (i.e., absolute magnitude estimation [19]). These magnitude estimations were normalized into z-scores, based on each participant's mean and standard deviation. Kinetic measures (the vectors orthogonal and normal to the grip on the transducer) were sampled at 1000 Hz from the force transducers to yield grip and load forces. The data from each transducer (representing each finger) were summed, passed through a dual pass 4^th^ order Butterworth filter with a low-pass cutoff of 14 Hz, and differentiated to yield grip force rate and load force rate. The maximum values from these measures represented the kinetic dependant variables. Participants lifted (or, in the no-vision task, saw) each cube 15 times, with one of three randomized lift orders (45 lifts in total). These data were then examined in separate 3 (cube size)×15 (trial) repeated measures ANOVAs.
